# The effect of disc-shaped gastric resection of anastomosis site on reducing postoperative dysphagia and stricture after esophagogastric anastomosis in patients with esophageal cancer

**DOI:** 10.1093/gastro/gow002

**Published:** 2016-02-17

**Authors:** Rahim Mahmodlou, Kamran Shateri, Faramarz Homayooni, Sanaz Hatami

**Affiliations:** 1 Department of Surgery, Urmia University of Medical Sciences, Urmia, Iran; 2 Department of Gastroenterology, Urmia University of Medical Sciences, Urmia, Iran; 3 Students’ Research Committee, Urmia University of Medical Sciences, Urmia, Iran

**Keywords:** esophageal cancer, esophagectomy, esophagogastic anastomosis, postoperative complications

## Abstract

**Background:** Esophagectomy remains the most reliable technique for managing esophageal cancer, but anastomotic complications including postoperative leak, ischemia and stricture negatively affect outcomes of this specific surgery. The aim of this study was to evaluate the effects of a novel method of esophagogastric anastomosis for reducing postoperative dysphagia and stricture formation.

**Methods:** Eighty patients who were scheduled for esophagectomy due to esophageal cancer were randomly assigned into two groups: intervention and control (40 each). In the control group, the esophagogastric anastomosis was performed with a linear gastric incision, whilst in the intervention group a new method of disc-shaped gastric resection for anastomosis was applied. Postoperative outcomes were compared between the two groups.

**Results:** The incidence of postoperative dysphagia and anastomotic stricture was significantly lower in the disc-shaped resection group (dysphagia 45% *vs* 75%, *P* = 0.02; stricture 12.5% *vs* 32.5%, *P* = 0.03), whilst the length of stay in an intensive care unit (ICU), anastomotic leakage and other complications were not significantly different between the two groups (all *P* > 0.05).

**Conclusion:** Anastomotic complications can be reduced by improving surgical techniques. The decreased incidence of postoperative dysphagia and anastomotic stricture in our study may be partly due to providing the proper diameter for the site of anastomosis when using the disc-shaped gastric resection method. Hence, this new method can improve the clinical outcomes of patients who undergo esophagectomy with esophagogastric anastomosis.

## Introduction

Esophageal cancer is one of the most frequent cancer-related causes of death worldwide [1], and the geographic area extending from northern Iran to north-central China has been considered a high-risk area for esophageal cancer (i.e. “esophageal cancer belt”) [2]. Surgical approach remains the most reliable modality for the management of esophageal cancer [3]. Anastomotic complications including postoperative leak, ischemia and stricture negatively affect outcomes of esophagectomy and may be indicators of the quality of anastomosis [4,5]. Development of such complications defeats one of the main palliative benefits of surgery, which is the relief of dysphagia [5–7]. Dysphagia is the principal complaint of patients who have anastomotic stricture [8] and is associated with subsequent malnutrition, dehydration, pneumonia and adverse health outcomes [9]. Stenosis occurs in 5–50% of operated cases within the first year after surgery [10].

Given the high frequency of postoperative stenosis and its devastating effects on the patient’s quality of life, several studies (using different methods such as hand-sewn or stapling techniques for anastomosis) have been undertaken to reduce the incidence of this complication, but the results of these studies have been controversial [7,11]. Considering the low rate of postoperative stenosis in colorectal anastomosis, in which resection is usually performed using a disc-shaped method on both sides, we decided to perform this study to investigate the effect of the disc-shaped resection method for esophagogastric anastomosis during esophagectomy in the development of postoperative dysphagia and anastomotic stricture.

## Methods

### Participants

During 2013, 80 patients with proven resectable esophageal cancer (stage III or lower) were enrolled in our study. All patients were diagnosed based on the findings of fibrogastroscopic biopsy. Patients were also assessed by barium meal tests and computed tomography (CT) scans, and fiberoptic bronchoscopy was performed in those patients with a tumor in the middle part of the esophagus to assess tracheal invasion. Patients with nonresectable tumors due to proven distant metastases, cervical esophageal tumors and tumor adhesion to the vital mediastinal organs such as trachea, malignant pleural effusion, poorly controlled diabetes, chronic kidney disease and chronic pulmonary disease were excluded from the study. The study patients were randomly divided into intervention and control groups (40 subjects each).

### Surgical procedure

**Figure 1.**
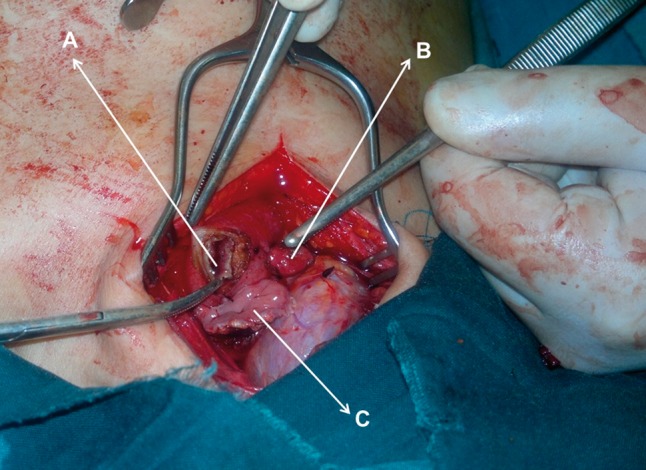
Intraoperative illustration of the disc-shaped resection of stomach (A) in our intervention group for the purpose of esophagogastric anastomosis. The shrunken resected section of the stomach (B) and the proximal remaining cervical esophagus (C) are shown in the picture as well

The general surgical approach in both groups was transhiatal esophagectomy (Orringer technique). Initially, the abdomen was entered through a midline supraumbilical incision and carefully examined for the presence of liver metastasis and ascites. The stomach was released as the conduit on the basis of the right gastroepiploic artery as described by Orringer *et al* [12–14]. In the next step, a 5–7 cm oblique incision was made along the anterior border of the left sternocleidomastoid muscle to the level of cricoid cartilage, and the cervical esophagus was detached and mobilized to the level of carina. In the third step, the hiatus was widened to allow the thoracic esophagus to be mobilized freely within the mediastinum. Then the tumor was resected with the majority of cervical esophagus, and only a short part of the cervical esophagus was preserved for anastomosis. Lower mediastinal lymphadenectomy was performed under direct vision as per routine. After complete mobilization of the stomach, the gastric conduit was formed with a linear stapler. Then the stomach tube was pulled up to the cervical wound and fixed to prevertebral fascia. The remaining esophagus was aligned with the stomach, and the site of anastomosis was selected. In the control group, the anastomosis was performed via linear incision in the midline wall of stomach as per routine with the Gambee pattern 3-0 silk sutures. In the intervention group, instead of performing a linear incision, we resected a round, disc-shaped part of the stomach with a diameter of 3–4 cm (with adequate distance from the stapled site of the stomach) and then completed the esophagogastric anastomosis with a Gambee-pattern suture (Figure 1). Right posterolateral thoracotomy was performed in two patients in the control group whose tumors were diagnosed as nonresectable via the hiatus. A #14 French rubber jejunostomy tube was inserted about 20–25 cm beyond the ligament of Treitz in all patients.

### Follow-up and assessment

Patients were followed and visited every month for the first six months after surgery and then every three months in the absence of dysphagia or other complaints. Patients with postoperative dysphagia underwent a barium meal test and endoscopic examination. The decision for endoscopic balloon dilatation was made based on the findings of the barium swallow study. Prior to any endoscopic dilatation (if needed) multiple biopsy specimens were taken, and patients were classified as having a benign stricture after recurrence of the malignancy had been ruled out. Dysphagia was scaled according to a modified version of the Dysphagia Severity Rating Scale (DSRS) [15]. We stratified the DSRS score to three levels: a score of 0 represented a lack of dysphagia, scores 1–3 were regarded as mild, and scores ≥ 4 were considered moderate to severe dysphagia. Hence, patients with change in sensation during swallowing, regardless of any increase in eating times and with or without any need for changes in diet, were considered to have mild dysphagia. Those who required oral supplemental nutrition or any other feeding intervention were defined as having moderate to severe dysphagia. The stricture was diagnosed according to the barium swallow assessment results. Patients were visited one week and one month after the surgery; in the case that they were symptom-free, routine visits were scheduled. All patients were followed for 9–12 months, which is the most common time window for developing anastomotic strictures.

### Statistical methods

The two groups were compared for the development of dysphagia, anastomotic strictures, leakage, wound infection, any pulmonary complication, ICU length of stay and recurrence of malignancy. The main endpoints were postoperative dysphagia and stricture formation. Data were expressed as mean ± standard deviation for continuous variables and frequency and percentage for categorical ones. The independent *t*-test was used to compare groups’ means, and the chi-square or Fisher exact test was used as needed. Data analyses were performed using SPSS software version 16 (Chicago, IL) and *P*-values < 0.05 were considered statistically significant.

## Results

Eighty patients with esophageal cancer were randomly assigned into either the intervention or control group (each 40) and underwent transhiatal esophagectomy. The chief complaints included dysphagia in all patients (100%), chest pain in 11 patients (27.5%) and weight loss and cachexia in 29 (72.5%) and 3 (7.5 %) patients, respectively. Patients’ characteristics are shown in Table 1.

**Table 1.** Patients’ characteristics

		Intervention (N = 40)	Control (N = 40)	P-value
Age (mean±SD, years)		62.10 ± 10.57	62.40 ± 10.73	0.90
Sex (male/female)		21/19	21/19	0.99
Chief complaint, n (%)	Dysphagia	20 (50)	20 (50)	0.99
	Heartburn	11 (27.5)	11 (27.5)	
	Weight loss	29 (72.5)	29 (72.5)	
	Cachexia	3 (7.5)	3 (7.5)	
Site of tumor, n (%)	Mid esophagus	14 (35)	9 (22.5)	0.37
	Lower esophagus	20 (50)	26 (65)	
	Cardia	6 (15)	5 (12.5)	
Pathology, n (%)	Adenocarcinoma	10 (25)	14 (35)	0.32
	SCC	30 (75)	26 (65)	
Involvement of the resection margin, n (%)		5 (12.5)	6 (15)	0.99

SD: Standard deviation; SCC: Squamous cell carcinoma;

In both groups, three patients (7.5%) had received radiotherapy prior to surgery. None of the patients in our study needed to undergo thoracotomy for major bleeding or damage to the trachea. In the intervention group, 28 patients (70%) stayed < 10 days, and 12 patients stayed > 10 days in the ICU. In the control group, 26 patients (65%) had < 10 days, and 14 had > 10 days in the ICU. There was no significant difference between the two groups with regard to ICU length of stay (*P* = 0.63).

In the intervention group, 10 patients (25%) had mild postoperative dysphagia, and eight (20%) suffered from moderate to severe dysphagia. In the control group, we observed mild dysphagia in 17 patients (42.5%) and moderate to severe dysphagia in 13 patients (32.5%). The frequency of postoperative dysphagia was significantly different between the two groups (45% *vs* 75%, *P* = 0.02). In the present study, no malignancy recurrence was observed in patients with postoperative dysphagia during the 9–12 months of follow-up. All patients with dysphagia were evaluated by the barium meal test and endoscopy, and five and 13 patients, respectively, from the intervention and control groups were diagnosed with anastomotic stricture requiring endoscopic dilatation. The rate of postoperative anastomotic stricture was significantly lower in the intervention group compared with the control group (12.5% *vs* 32.5%, *P* = 0.03). The rate of anastomotic leakage, wound infection and pulmonary complications are listed in Table 2. No significant difference was observed between the two groups regarding these complications (all *P*> 0.05).

**Table 2.** Postoperative outcomes

		Intervention (N = 40)	Control (N = 40)	*P*-value
Postoperative dysphagia	No dysphagia	22 (55)	10 (25)	0.02
	Mild	10 (25)	17 (42.5)	
	Moderate to severe	8 (20)	13 (32.5)	
Postoperative complications	Anastomotic leakage	7 (17.5)	5 (12.5)	0.33
	Anastomotic stricture	5 (12.5)	13 (32.5)	0.03
	Wound infection	9 (22.5)	7 (17.5)	0.40
	Pneumonia	11 (27.5)	10 (25)	0.71

## 

## Discussion

Anastomotic complications including postoperative leakage, ischemia and stricture negatively affect the outcome of esophagectomy [4,5]. Different surgical techniques have been shown to be associated with different levels of complication, and surgeons and researchers are trying to minimize these procedure-related complications [16,17]. The mechanism for developing anastomotic complications such as leakage or stricture is not fully understood [3,18]. In addition to the site of anastomosis, many factors including tension on the anastomotic site, the site’s arterial blood supply and venous circulation, the surgeon’s expertise along with the method of anastomosis can influence the risk of anastomotic complication. Performing a tension-free anastomosis with adequate blood supply has been suggested to prevent subsequent stricture formation. The rate of anastomotic stricture varies across different institutions and different studies in the literature and ranges from 0% to 21% for the circular stapled anastomosis method across different studies [19–24]. The noticeable variation in reported rates of anastomotic stricture may also be due to lack of a widely accepted definition for postoperative anastomotic stricture [8].

In this study we tested disc-shaped gastric resection versus linear incision of the stomach at the site of anastomosis to reach the optimum hollow conduit needed for swallowing. The rate of postoperative dysphagia and stricture was significantly lower in the intervention group, whilst the prevalence of anastomotic leak and other complications (pulmonary complications or wound infection), mortality rate and ICU length of stay were not statistically different between groups. This lower rate of dysphagia and stricture could be attributed to a larger surface area for the site of anastomosis in the disc-shaped (circular) method compared with the linear method. Two studies have investigated the postoperative outcomes of circular (or disc-shaped) versus linear gastric incisions for esophagogastrostomy. They have shown better performance for the linear anastomosis [23,24], but this lower rate of anastomotic stricture seemed to be gauged by a higher anastomotic diameter in the linear arms of those studies rather than the superiority of linear method over circular one. For example, Xu *et al.* compared a group of 166 patients who underwent esophagogastrostomy with the linear stapled method with 68 subjects who underwent the same surgery with the circular stapled technique and demonstrated a significantly higher rate of anastomotic stricture in the circular stapled group compared with the other linear stapled group (20.9% *vs* 1.9%; *P* < 0.001) [24]. This could be related to a significantly higher mean diameter of the anastomotic orifice in the linear group compared with the circular arm (1.6 *vs* 1.0 cm). This interpretation is consistent with the findings of Petrin *et al.* who showed the incidence of anastomotic stricture to be inversely related to the diameter of stapler [22]. In another study, Collard *et al.* suggested that an anastomosis area > 125 mm^2^ (∼1.25 cm of anastomotic orifice diameter) is required for easy swallowing of solid foods [25].

Many institutions prefer to use the total mechanical anastomosis technique (linear or circular stapler) to simplify the operation and reduce the operation time [20,25,26]. It should be mentioned that use of the linear stapler is limited to performing cervical esophagogastric anastomoses because of its large size. The circular staplers, on the other hand, are not available in the majority of developing countries such as Iran (due to issues such as higher cost and the need for technically trained staff). Hence, in this study we decided to perform hand-sewn, single-layered Gambee pattern sutures for anastomosis, as we do routinely for transhiatal esophagectomy.

Reports are conflicting about the optimal method of anastomosis (hand-sewn *vs* stapler) [6,7,21,27]. In a systematic review, Kim *et al.* have shown no difference between the post-operative anastomotic leakage rates of the two above-mentioned anastomosis techniques (i.e. hand-sewn *vs* stapled) [7]. Among five studies that have evaluated postsurgery anastomotic strictures, only one study has reported a significantly lower rate of stricture in the hand-sewn method compared with the stapled method [28]. These findings were confirmed by another meta-analysis, done recently by Wang *et al*., that showed no significant difference in the risk of developing anastomotic leak or stricture between hand-sewn and stapled esophagogastric anastomosis techniques [27]. Nozaki *et al.* reported the complication rates of hand-sewn anastomoses and concluded that they were comparable to the complication rates of mechanically stapled anastomoses [29].

As mentioned above, we evaluated all patients with the postoperative complaint of dysphagia (regardless of the severity: mild, moderate or severe) with a barium meal test as well as endoscopic examination where needed and observed that five (27.7%) of the 18 patients in the intervention arm with postoperative dysphagia had a stricture that required endoscopic dilation. The rate was higher in the control group (13 cases, 43.3% of the 30 patients with postoperative dysphagia).

Although increased anastomotic area size via the above-mentioned surgical method may help to reduce the postoperative stricture rate as shown in this study, it may raise the likelihood of reflux, which was not assessed in this specific trial. Further studies taking a broader spectrum of complications into account, as well as following-up a larger cohort of patients for a longer period of time, are warranted.

In conclusion, we showed that disc-shaped (circular) gastric resection during esophagogastric anastomosis may lead to a lower rate of postoperative dysphagia and anastomotic stricture compared with the former linear incision method. This method can significantly improve the clinical outcome for patients who undergo transhiatal esophagectomy and may improve their postoperative quality of life.
